# Prolonged survival in secondary glioblastoma following local injection of targeted alpha therapy with ^213^Bi-substance P analogue

**DOI:** 10.1007/s00259-018-4015-2

**Published:** 2018-04-30

**Authors:** Leszek Krolicki, Frank Bruchertseifer, Jolanta Kunikowska, Henryk Koziara, Bartosz Królicki, Maciej Jakuciński, Dariusz Pawlak, Christos Apostolidis, Saed Mirzadeh, Rafał Rola, Adrian Merlo, Alfred Morgenstern

**Affiliations:** 1 Department of Nuclear Medicine, Medical University of Warsaw, ul. Banacha 1 a, 02-097 Warsaw, Poland; 2 European Commission, Joint Research Centre, Directorate for Nuclear Safety and Security, Karlsruhe, Germany; 3 Department of Neurosurgery, Institute of Psychiatry and Neurology, Warsaw, Poland; 4 Department of Nuclear Medicine, Brodnowski Hospital, Warsaw, Poland; 5 Radioisotope Centre POLATOM, National Centre for Nuclear Research, Otwock, Poland; 6 Nuclear and Radiochemistry Group, Nuclear Security and Isotope Technology Division, Oak Ridge National Laboratory, Oak Ridge, TN 38731-6229 USA; 7 Department of Neurology, Military Institute of Aviation Medicine, Warsaw, Poland; 8 Neurosurgery, Bern and University of Basel, Spitalgasse 32, Bern, Switzerland

**Keywords:** Glioblastoma, GBM, ^213^Bi-DOTA-SP, ^68^Ga-DOTA-SP, Targeted alpha therapy, Substance P

## Abstract

**Background:**

Glioblastoma multiforme (GBM), the most common malignant brain tumor, mainly manifests as a primary de novo and less frequently as a secondary glial neoplasm. GBM has been demonstrated to overexpress the NK-1 receptor and substance P can be used as a ligand for targeted therapy. Alpha emitters, e.g. ^213^Bi, that deposit their high energy within a short range allow the selective irradiation of tumor cells while sparing adjacent neuronal structures.

**Material and methods:**

Among 50 glioma patients of different subtypes that have to date been treated with targeted alpha therapy at the Medical University Warsaw, we report here the data on nine patients with secondary GBM. Following surgery, chemo- and radiotherapy, recurrent GBM was treated by intracavitary injection of 1–6 doses of 0.9–2.3 GBq ^213^Bi- DOTA-[Thi^8^,Met(O_2_)^11^]-substance P (^213^Bi-DOTA-SP) in 2-month intervals. ^68^Ga-DOTA-[Thi^8^,Met(O_2_)^11^]-substance P (^68^Ga-DOTA-SP) was co-injected with the therapeutic doses to assess biodistribution using PET/CT. Therapeutic response was monitored with MRI.

**Results:**

Treatment with activities ranging from 1.4 to 9.7 (median 5.8) GBq ^213^Bi- DOTA-SP was well tolerated with only mild transient adverse reactions, mainly headaches due to a transient perfocal edema reaction. The median progression free survival and overall survival time following the initiation of alpha therapy was 5.8 and 16.4 months, respectively. The median overall survival time from the first diagnosis was 52.3 months. Two out of nine patients are still alive 39 and 51 months, respectively, after the initiation of the therapy.

**Conclusions:**

Targeted alpha therapy of secondary GBM with ^213^Bi-DOTA-SP is safe and well tolerated and may evolve as a promising novel therapeutic option for secondary GBM.

## Introduction

Glioblastoma multiforme (GBM) is the most common malignant primary brain tumor which evolves either as a primary de novo GBM or as a transformed secondary GBM out of a less malignant precursor lesion. Primary GBMs develop rapidly in more elderly patients without histologic evidence of precursor elements whereas secondary GBMs, which account for about 9% of all grade IV tumors, progress from low-grade or anaplastic astrocytoma [1].

Standard treatment of glioblastomas consists of debulking surgery followed by radio- and chemotherapy [2–4]. Although a variety of chemotherapeutic agents has been evaluated for this indication, response rates were observed in the range of 0 to 20% [4–6]. Similarly, classical radiotherapy is limited to a maximum dose of 60 Gy applied in daily 2 Gy fractions, as recommended by the Radiation Therapy Oncology Group (RTOG), due to the toxicity of the adjacent normal brain tissue. The addition of temozolamide chemotherapy to standard external beam radiotherapy led to a mean survival of 14.6 months, as compared to 12.1 months with radiotherapy alone [3]. However, this more aggressive combination of therapies has some limitations, e.g. severe fatigue, thromboembolic events or opportunistic infections of mainly lung tissue [7–9]. Only about half of the patients displaying methylation of the O^6^-Methylguanine-DNA methyltransferase (MGMT) gene benefit [10–12].

To enhance the intratumoral drug concentration, local drug delivery has been proposed to circumvent the blood brain barrier [13, 14]. This way, the therapeutic agent is injected directly into the resection cavity or into the tumor mass via a dedicated port-a-cath-system. An increased intratumoral pressure may counteract local drug distribution, and specific preparations have to be undertaken to control pressure levels, e.g. intravenous administration of an osmodiuretic drug and insertion of more than one port-a-cath system [15]. This way, the locally administered active substance generally will be distributed more homogeneously throughout the tumor mass and finally destroy the tumor upon repetitive local injections.

To execute targeted therapy in nuclear medicine, suitable receptors have to be identified which are specifically overexpressed in the tumor. Among several vectors that have been identified and clinically tested for targeting of glioma [14–18], the peptidic vector substance P with low molecular weight has been proven useful. Substance P binds to the neurokinin type 1 receptor (NK-1) which is consistently overexpressed in all gliomas regardless of the degree of malignancy [15, 19]. Therefore, substance P analogues are considered to be the best candidates for intralesional treatment of glial neoplasms.

In targeted radiotherapy using radiolabeled vectors, the therapeutic effect critically depends on the type of source used, in particular on energy and range. Both parameters play a key role in the radiobiological process that leads to tumor cell death. Two types of radioisotopes are mainly being used, β^−^-emitting isotopes: ^131^I, ^90^Y, ^177^Lu; and α-emitting isotopes: ^225^Ac, ^213^Bi, ^211^At. For brain tumor treatment, neuronal toxicity can be best controlled by ultrashort range α emitters [20]: the short range of α particles of less than 100 μm corresponding to only a few cell diameters allows selective irradiation of tumor cells. Therefore, glioma cells invading functionally critical areas of the brain can be targeted and destroyed [20]. The very high energy of several MeV released by an α decay primarily induces DNA double strand breaks, a mainly irreparable DNA damage, that minimizes sublethal damage and leads to cell death.

This paper presents the results of targeted alpha therapy (TAT) with radio-labelled ^213^Bi-DOTA-Substance P analogue in nine patients with secondary GBM. The primary end point was the feasibility and toxicity of the approach. The secondary end point was the outcome defined as the progression free survival (PFS) and overall survival (OS).

## Patients and methods

Fifty patients with different malignant glioma tumors were included into the study at the Medical University of Warsaw. Secondary GBM was diagnosed in seven female and two male patients (age range 21–59 years, mean 38.8 ± 10.8 years) out of these 50 patients. Tumors were located in the frontal lobe in five patients, in the parietal lobe in three patients and in the temporal lobe in one patient.

Clinical and histopathological criteria were used to distinguish between glioblastoma subtypes: primary GBM was histopathologically defined as grade IV lesion at the first biopsy, without clinical or histopathologic evidence of a less malignant precursor lesion. The diagnosis of secondary GBM was made only in cases with histopathologic evidence of preceding low-grade or anaplastic astrocytoma. All patients previously underwent surgery with primary histopathological examination: diffuse astrocytoma grade II in six patients, anaplastic astrocytoma grade III in two patients, in one patient conversion first from grade II to III and after 2 years to grade IV was diagnosed.

Clinical history of patients revealed epilepsy and focal neurological symptoms like paresis, aphasia and visual impairment as a result of tumor location. All patients were treated with steroids and anti-epileptic drugs.

The study was approved by the Ethical Committee of the Medical University of Warsaw. Written informed consent was obtained from all patients. The following inclusion criteria were used:

- Histologically confirmed secondary astrocytoma grade IV (WHO)
- Tumor volume below 90 mL as defined by the T1-weighed contrast-enhanced MRI
- Non-evidence for obstruction of CSF circulation or decompensating intracranial pressure
- Karnofsky performance score > 40
- No pregnancy or lactation
- Age older than 18 years; absence of psychological, familial, sociological conditions potentially hampering compliance with the study protocol.

### Production of ^225^Ac/^213^Bi and radiolabelling

^225^Ac was produced by radiochemical extraction from a ^229^Th source at the Directorate for Nuclear Safety and Security of JRC Karlsruhe [21] and in some instances also at Oak Ridge National Laboratory [22]. The activity was pooled and was loaded on a ^225^Ac/^213^Bi radionuclide generator using AG MP-50 cation exchange resin (Bio-Rad) [23]. ^213^Bi was eluted at intervals of 2–3 h using 1.4 mL 0.1 M HCl/0.1 M NaI. The labelling of DOTA-[Thi^8^, Met(O_2_)^11^]-substance-P was performed in 2 M TRIS buffer using a microwave synthesizer (Biotage® Initiator) at 95 °C for 5 min. Quality control was performed by instant thin-layer chromatography (Tec Control 150–771; Biodex Medical Systems). Radiochemical purity was higher than 99%.

Eluates of 5 mL volume from the ^68^Ge/^68^Ga generator (iThembaLABS, Republic of South Africa) with radioactivity of 350–800 MBq of ^68^Ga were used for labeling of 150 μg DOTA-[Thi^8^, Met(O_2_)^11^]-substance-P (piChem, Austria) dissolved in 2.4 mL 1.25 M sodium acetate, reaching a final pH of ~3.9. Incubation was carried out at 100 °C for 10 min using a conventional heating plate. Radiochemical purity was assessed by ITLC-SG chromatography with 0.05 M sodium citrate as solvent. Products with radiochemical purity higher than 95% were used for preparation of diagnostic doses; 10 MBq of ^68^Ga in a volume of ≤0.5 mL (typically 0.1–0.2 mL) of ^68^Ga-DOTA-SP were added to the therapeutic dose of ^213^Bi-DOTA-SP.

Before injection, sterility of the final formulation was ensured via sterile filtration (Millex-GV, 0.20 μm; Millipore). Injections were performed typically 15 ± 2 min after the end of ^225^Ac/^213^Bi generator elution.

### Study protocol and injection of the radiopharmaceutical

**Fig. 1:**
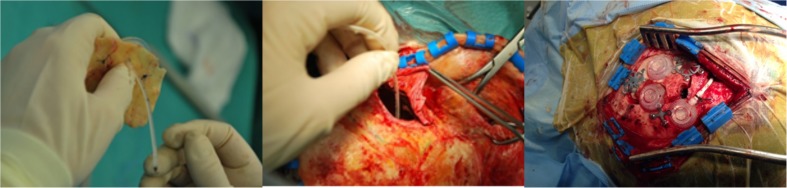
Implementation of catheters into the postsurgical cavity. A cavity of 1.5 cm diameter is drilled into the external *tabula* of the skull with a central opening. A port capsule connected with a catheter is then stereotactically inserted into the tumor or resection cavity. The wound is then closed. Injections into the capsule were performed some 10 days later

A standard neurosurgical ventricular catheter connected to a subcutaneous port (Medtronic) was stereotactically implanted either into the postsurgical cavity or intratumorally 2–4 weeks before treatment (Fig. 1). To confirm the correct catheter position and to exclude any connection with the CSF system, a test injection was performed using a 1:20 gadolinium-saline solution.

During ^213^Bi-DOTA-SP therapy, corticosteroids and antiepileptic drugs were continued. Immediately before injection of the radiopharmaceutical, 60 ml of mannitol 10 Vol% and 12 mg of dexamethasone (Fresenius, PharmaSwiss) were applied for transient reduction of intracranial pressure.

For local injection of the radiopharmaceutical, the port was punctured with a butterfly needle. Following a control injection of 1 mL of saline, the active drug was injected in the volume of 2 mL. Then, the system was flushed again with 1 mL of saline. The injection velocity was 0.5 mL/min. For each therapeutic cycle, 1–3 injections of up to 1.8 GBq per injection were performed depending on the activity obtained from the ^225^Ac/^213^Bi radionuclide generator. Patients were treated with 1–6 cycles of ^213^Bi- DOTA-SP in 2-month intervals.

A preparation of 10 MBq of ^68^Ga-DOTA-SP was co-injected with the therapeutic doses to assess biodistribution using PET/CT. PET/CT was performed 30 min after each injection to confirm orthotopic intralesional injection. Blood samples were taken 15, 30 and 45 min post injection to assess systemic distribution of the radiopharmaceutical.

### ^68^Ga-DOTA-SP PET/CT protocol

A three-dimensional mode was used for PET/CT examinations performed on a Biograph 64 TruePoint PET/CT scanner (Siemens Medical Solutions, Knoxville, TN), 30 min after the co-injection of ^68^Ga/^213^Bi –DOTA-SP. Five minutes acquisition time was used for PET of brain and 2 min acquisition per bed position for body (from the neck to the upper thighs).

The PET image data were corrected for scatter and attenuation using the CT data and reconstructed in a 168 × 168 matrix. The reconstruction was performed using the TrueX algorithm (Siemens Medical Solutions) with PSF, three iterations, 21 subsets, and no post filtering.

The PET/CT images (half-body-attenuated and non-attenuated PET, CT, and fused images) were transferred to a multimodal work station (MMWS; Syngo TrueD; Siemens Medical Solutions) for analysis.

### MRI evaluation

All patients underwent MRI examination (GE Excite HD, 1.5 T). T1 before and after gadolinium injection, T2, FLAIR and DWI sequences were performed before and every 6 weeks after each cycle of therapeutic doses of ^213^Bi-SP and in a 2–3 month interval follow up after treatment.

For the evaluation of tumor response, the Revised Assessment in Neuro-Oncology (RANO) criteria were used [24, 25]. The progression of the disease was defined as follows: 25% or more increase in enhancing lesions despite stable or increasing steroid dose, increase in non-enhancing T2/FLAIR lesions, not attributable to other not tumor-related causes; any new lesions, or clinical deterioration (not attributable to other not tumor-related causes and not due to steroid decrease) were observed.

Quantitative tumor assessment was performed by radiologists with at least 5 years of experience. The tumor volumes were analyzed by defining the tumor extension on the T1-weighed gadolinium enhanced and on the T2-weighed MRI. Tumors were manually outlined on digitized image data using Olea Sphere software (Olea Medical).

### Statistical methods

Parameters were characterized by the median or mean (± SD). The progression free survival (PFS) was defined as the time from the start of radioisotope treatment to the first evidence of progression or relapse, or to death. The overall survival from the diagnosis (OS-D) was defined as the time from the first diagnosis of the tumor to death from any cause; the OS from conversion (OS-C) was determined as the time from a histologically proven conversion to a grade IV tumor to death from any cause; and the OS from the start of treatment (OS-T) was defined as the time from the first cycle of ^213^Bi-DOTA-SP treatment to death from any cause.

The OS and PFS were calculated using the Kaplan-Meier estimator and compared using the log-rank test. Calculations were performed using GraphPad PRISM 5 (GraphPad Software Inc).

## Results

### Patient characteristics and functional status

Before targeted alpha therapy, the median Karnofsky status was 80 (range, 40–100) (Table 1). The functional status was assessed using the Barthel Index which evaluates neurological function and impairment in activities of daily living. The median pre-therapeutic Barthel index was 70 (range, 45–100).

**Table 1:** Patient characteristics

Patient number	Patient initials	Age (years)	Conversion/ Grading	Time to conversion [months]	Tumor volume [ml]	Karnofsky status	Barthel status	Number of courses	Total activity [GBq]	PFS [months]	OS-t [months]
1	ZA	42	II/III/IV	53.5	3.9	80	60	4	7.6	3.9	16.4
2	BA	38	II/IV	12.9	15.6	80	100	3	5.8	23.8	27.0
3	SK	59	II/IV	18.4	60.9	60	70	2	4.2	1.7	9.9
4	ML	35	II/IV	13.2	81.5	40	50	2	3.1	2.1	2.9
5	KP	29	II/IV	37.6	57.5	90	100	6	9.7	13.6	23.7
6	S-GM^a^	42	II/IV	23.3	17.3	60	70	4	6.7	51.5	51.5
7	BK^a^	32	III/IV	10.6	42.3	100	100	4	8.0	39.2	39.2
8	KL	51	II/IV	10.7	64.3	50	45	1	1.4	0.3	2.9
9	MP	21	III/IV	38.7	18.7	90	100	2	3.1	5.8	8.5

^a^Still alive

These parameters were evaluated during therapy and the follow up period. At the last course of therapy, the median Karnofsky status was 70 (range, 30–100) and Barthel Index 65 (range, 40–100). Three months after the last course, the Karnofsky status was 70 (range, 30–100) and the Barthel Index 65 (range, 40–100). According to clinical status, patients were treated with 1–6 cycles of ^213^Bi-DOTA-SP in 2-month intervals. The median total injected activity was 5.8 GBq (range, 1.4–9.7 GBq).

### Post therapeutic imaging with ^68^Ga-DOTA-SP

**Fig. 2:**
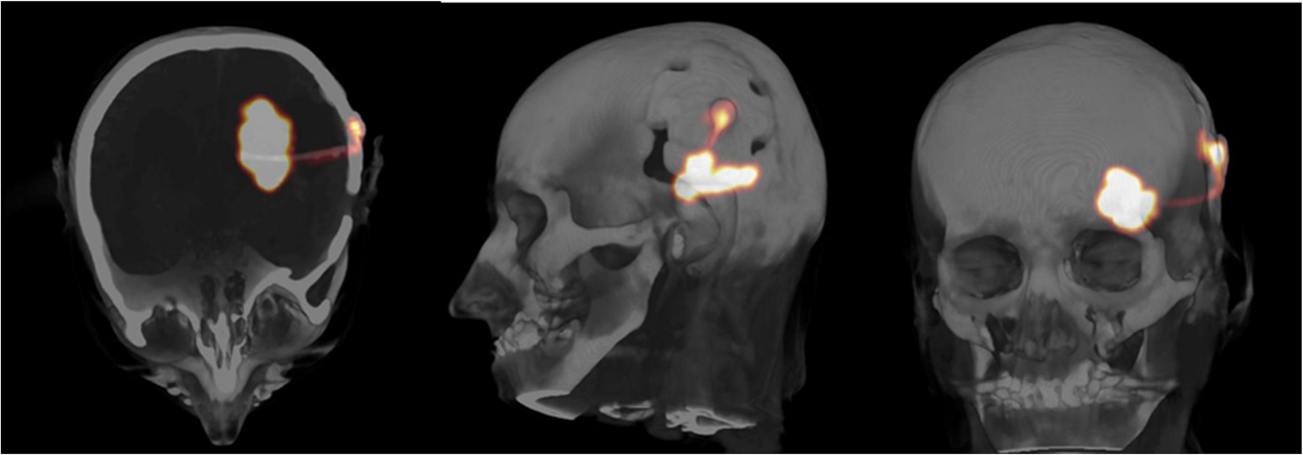
PET/CT after local co-injection of 10 MBq ^68^Ga-DOTA-SP with a therapeutic dose of ^213^Bi-DOTA-SP into the resection cavity of a glioblastoma in patient 2. Most of the activity is concentrated within the lesion, with little activity remaining in the capsule and within the catheter

**Fig. 3:**
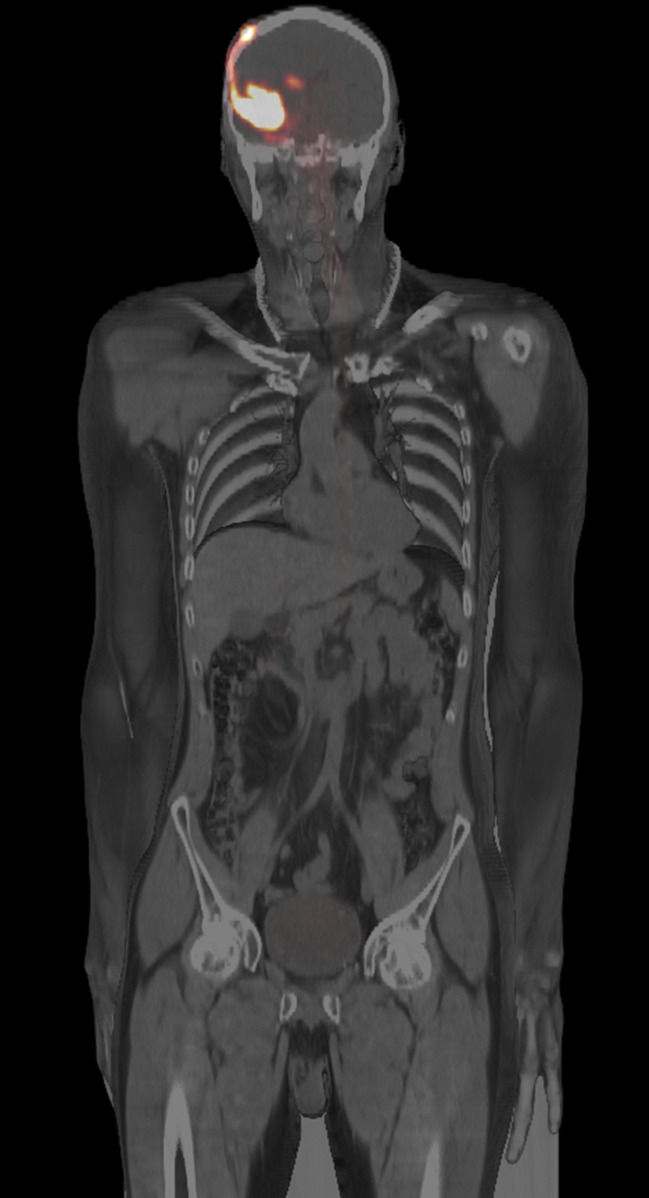
Whole body PET/CT scan shows biodistribution 30 min after intralesional injection of 10 MBq ^68^Ga-DOTA-SP analogue: the signal detected in the body outside the brain is very faint or negligible in liver, kidney, spleen and bone marrow. The cleaved linear peptidic vector is excreted into the bladder and can show a weak signal corresponding to <5% of injected activity

Post therapeutic scans were performed to evaluate tracer whole body biodistribution and expression of NK-1 receptor at the target site. In all cases, the uptake in the tumor area was increased as illustrated in a typical PET/CT image in Fig. 2. The whole body scans presented very low distribution in kidney and urine, with less than 5% of activity in the bladder (Fig. 3). Accordingly, ^213^Bi activities found in the blood pool typically corresponded to less than 8% I.D. and peaked at 1 h post injection.

### Side-effects of treatment with ^213^Bi-DOTA-SP

The local treatment of ^213^Bi-DOTA-SP was well tolerated. In only two patients, mild flush of the face was observed as a systemic SP effect when a small amount of the tracer was absorbed in the blood. In one patient, transient worsening of paresis and aphasia were observed for 5 days. No other severe adverse events occurred.

### Therapeutic outcome

**Fig. 4:**
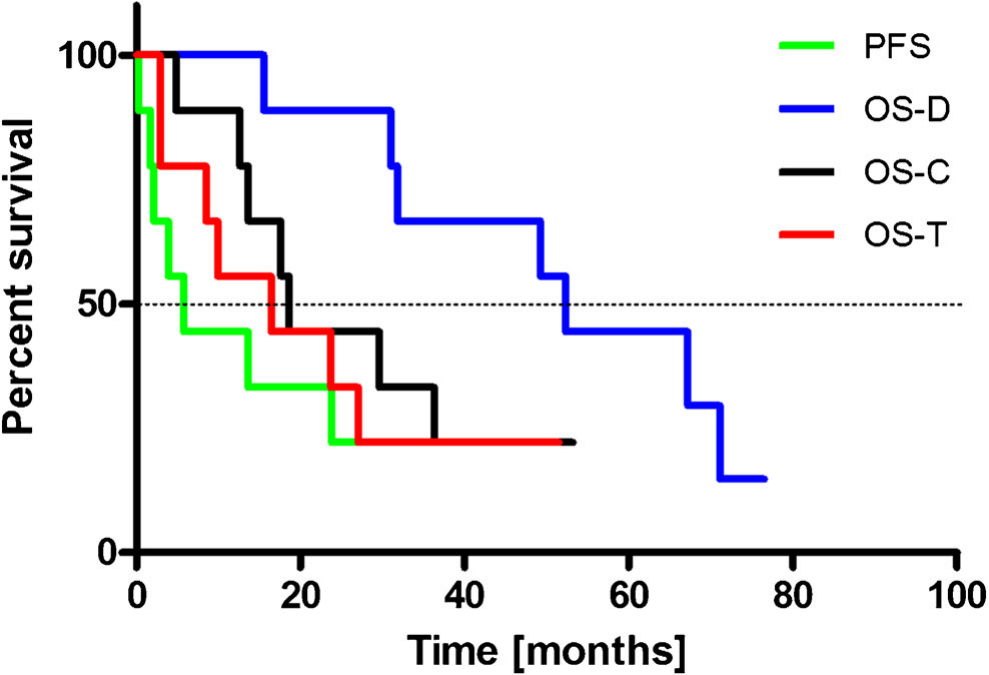
The Kaplan-Meier estimator displays the progression free survival (PFS), overall survival following initial diagnosis (OS-D), overall survival following conversion (OS-C) and overall survival after initiation of therapy with ^213^Bi-DOTA-SP analogue (OS-T)

The median time from the first diagnosis to the tumor transformation into GBM was 18.4 months (range, 10.6–53.5 months). The median overall survival time from the first diagnosis (OS-D) was 52.3 months; the median overall survival from conversion (OS-C) was 18.6 months. From the onset of ^213^Bi-DOTA-SP analogue treatment, the median PFS was 5.8 months and the OS-T was 16.4 months. The results are summarized in the Kaplan-Meier estimator curve (Fig. 4).

The MRI of the brain demonstrated postoperative changes with surgical margin enhancement and diffuse T2 and FLAIR changes consistent with edema and/or non-enhancing neoplasm.

The median total tumor volume (T1Vol), defined as solid tumor parts, resection cavity and putative necrotic areas on the T1-weighed gadolinium enhanced MRI images, was 42.3 mL (range, 3.9–81.5 mL). Further imaging analysis disclosed the median volume of solid tumor elements contrasted by gadolinium (T1VolCE) to be 9 mL (range, 1.0–50.6 mL), and necrotic areas plus resection cavity (T1VolNec) to be 15.1 mL (range, 3.9–73.3 mL). On the T2 images, the median volume of the pathological signal (T2Vol) was 165.9 mL (range, 29.1 and 320.2 mL).

Changes in tumor volumes were analyzed 2–3 months after the last dose of ^213^Bi-DOTA-SP analogue. T1Vol decreased to 29.7 mL (range, 11.6–136.9 mL), T1VolCE increased to 11.6 mL (range, 1.1–50.6 mL), and T1VolNec increased to 17.8 mL (range, 1.0–112.5 mL) and finally T2Vol decreased to 157.9 mL (range, 25.1 and 320.2 mL). These alterations in tumor volumes, however, did not reach statistical significance.

**Fig. 5:**
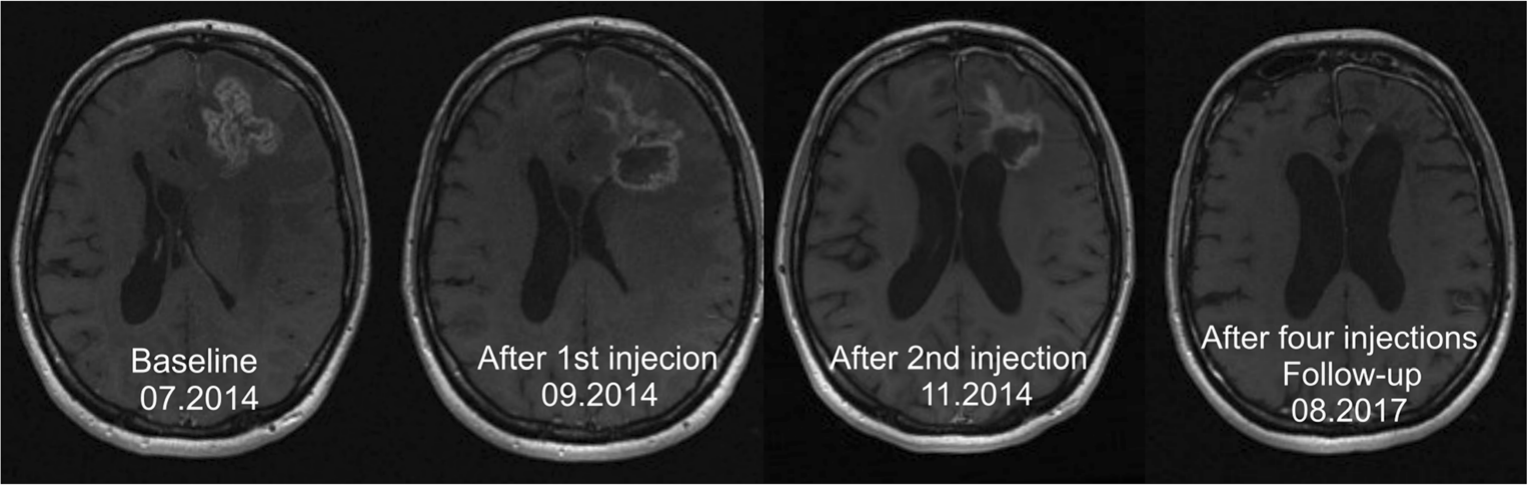
In a 32-year-old woman suffering from an astrocytoma WHO grade II, conversion into a secondary GBM manifested 10.6 months after initial diagnosis. Following standard treatment consisting of surgery, radio- and chemotherapy with TMZ, four cycles of ^213^Bi-DOTA-SP were applied. The total activity injected amounted to 8.0 GBq of the therapeutic isotope. The T1-weighted enhanced MRI examination revealed shrinkage of the tumor by 32%

An example of the therapeutic effect obtained by targeted alpha therapy in patient 7 is shown in Fig. 5. Follow-up of therapeutic responses and toxicity is continued and patient recruitment is ongoing.

## Discussion

Glioblastoma multiforme is the most frequent malignant brain tumor in adults which manifests either as rapidly growing de novo lesion or as a secondary GBM evolving out of a low grade or an anaplastic astrocytoma precursor lesion. Therefore, secondary GBM manifest in middle-aged individuals around the age of 45 years, while primary GBM occur in older patients with a median age of 62 years. Secondary GBM are more frequently found in females, and primary GBM more frequently in males [1]. Differences in survival times between both groups are controversial since age-adjusted multivariate analysis did not show a significant difference. However, log-rank tests showed that patients with primary GBM had a significantly shorter survival time than those with secondary GBM [26].

A widespread glioma cell infiltration into normal adjacent brain areas, the molecular heterogeneity as well as the protective effects of the blood-brain barrier are the main causes for the limited efficacy of front line standard treatments of GBM such as surgery, radio- and chemotherapy. Despite some minor improvements in the PFS, no obvious increase in the survival has been associated with any particular regimen. Only 25% of patients with progressive or recurrent GBM can be considered for repeat surgery. Re-irradiation remains a palliative option only for a selected group of patients characterized by the KPS > 60%, the tumor size <40 mm and progression for more than 6 months. Temozolamide (TMZ) monotherapy or TMZ-containing combinational therapies are controversial [7, 27]. Since therapeutic options for GBM are very limited, especially for patients with recurrent disease, novel treatments are urgently needed.

Regarding the genetic tumor classification, both tumor types develop through different genetic pathways and show different protein expression profiles with the main differentiating factor being IDH1 gene mutations in secondary GBM [1] observed at a frequency of 73% as compared to only 3.7% in primary glioblastomas [1, 28]. The IDH1-mutation is associated with a hypermethylation phenotype, and IDH1 mutations are the earliest detectable genetic alterations in precursor low-grade diffuse astrocytomas and in oligodendrogliomas. These data suggest that primary and secondary GBM may require different therapeutic approaches. However, these results show that local treatment of secondary GBM by a targeted alpha-labeled peptidic vector also represents a therapeutic option for this subtype of glioma.

Local application of the drug has major advantages: the intratumoral concentration of the drug is significantly higher in comparison to systemic treatment, the blood-brain barrier can be circumvented, and adverse reaction events are more limited. The main challenge of the local drug application into a brain tumor is the tumor saturation and, at least for larger targeting vectors, slow diffusion into the tumor. Currently, different strategies for local treatment are in development, like monoclonal antibodies, interleukins receptors, bacterial toxins, viruses, oligonucleotide CpG as well as radiopharmaceuticals [14, 17, 29–35].

The radiopharmaceuticals are very promising given the low molecular weight of the targeting vector <2000 Da, facilitating a rapid intratumoral drug distribution, and radiolabeling with an α-emitting isotope that releases a very high energy concentrated to a few tumor cells, precluding sublethal tumor cell damage. The peptidic vector substance P analogue used in this study is an eleven-amino acid neurotransmitter that appears in both the central and peripheral nervous systems. Autoradiography disclosed overexpression of neurokinin type 1 receptor in 95% of gliomas of WHO grades II, III and IV. NK-1-receptors have also been detected in tumor cells infiltrating the intratumoral and peritumoral vasculature [19].

The first clinical study with a radiolabeled DOTA-substance P analogue showed disease stabilization or improved neurologic status in 13 of 20 patients [15]. In most patients, the β^−^-emitting radionuclide Y-90 had been used, and the alpha-emitter Bi-213 was only available for two patients. In a further report, a pilot study with local injections of ^213^Bi-DOTA-SP in five patients with critically located gliomas, confirmed the initial experience that alpha therapy is well tolerated [20]. Likewise, the application of the radiopeptide was also well tolerated in our study on nine secondary GBM patients. Repeated MRI was suggestive of radiation-induced necrosis and demarcation of the tumors, and this finding was validated by the subsequent resection. Therefore, the study concluded that targeted local radiotherapy with ^213^Bi-DOTA-substance P analogue may represent an innovative and effective treatment strategy for critically located malignant gliomas, because primarily non-operable gliomas may become resectable over the course of treatment.

In patients with disease progression, we observed the median PFS of 5.8 months, the median OS from the first diagnosis of 52.3 months and from the start of ^213^Bi-DOTA-SP of 16.4 months. These results compare favorably with those reported in the literature where median overall survival rates of patients with secondary GBM who underwent partial or complete surgical resection were 7.8 and 2.5 months, respectively, in unresected cases; the OS of patients who received radiotherapy was 10 months and patients who did not receive radiotherapy 2.0 months [26].

The analysis of patients with glioblastoma with and without IDH1mut who were treated with surgery and radiotherapy showed that the OS time was 27.1 and 11.3 months, respectively [28]. From a radiobiological point of view, an application interval of 8 weeks in an exponentially proliferating tumor system using ^213^Bi with a short half-life of 46 min is less than optimal, nonetheless, the first experimental data obtained with this new approach suggest that the proposed local treatment of secondary GBM may become an effective therapeutic option.

A critical point in the local administration of radiopharmaceuticals is the control of the injection procedure to achieve maximum intratumoral biodistribution saturating not only the nodular tumor component, but also those glioma cells invading adjacent normal brain tissue. In previous studies with ^213^Bi-DOTA-SP, biodistribution was monitored by SPECT/CT, using the 440 keV γ-ray emission of ^213^Bi [15] requiring long acquisition times of about 30 min with limited resolution. In this study, ^68^Ga-DOTA-SP analogue was used for diagnostic PET/CT-imaging which was co-injected with therapeutic doses of ^213^Bi-DOTA-SP. This allowed monitoring of the drug distribution in the brain and in the whole body with PET/CT with acquisition times of 5 min (brain) and 10–12 min (whole body), respectively. The whole body scans presented very low distribution outside the brain, with less than 5% of injected activity in the bladder.

There are two limitations to this study. First, the number of patients is small, since GBM primarily manifest as de novo lesions. Nevertheless, the OS-T was longer in this study than reported in the literature after surgery, radio- and chemotherapy alone. Secondly, no genetic examinations have been performed to disclose IDH1 mutations manifest in 73% of secondary GBM [28]. To corroborate these data, more extensive studies with a larger number of patients are required.

Overall, our results indicate that local injection of ^213^Bi-DOTA-SP represents a promising new strategy for therapy of secondary GBM. From a technical perspective, its implementation in clinical practice does not pose particular challenges. ^225^Ac /^213^Bi radionuclide generators can be safely handled using established procedures and standard equipment typically available in nuclear medicine departments. However, for a widespread clinical application, the supply of ^225^Ac as mother nuclide for ^225^Ac/^213^Bi generators must be significantly increased beyond existing limited levels. Accelerator-driven processes that allow its production on a large scale have been developed [36] and need to be implemented to overcome these supply limitations.

## Conclusions

Treatment of secondary GBM with ^213^Bi-DOTA-SP analogue is safe and well tolerated. This targeted alpha therapy may evolve as a new option for the treatment of secondary GBM, provided that more extensive studies confirm these initial findings and the supply of ^225^Ac as a source for ^213^Bi is secured.
